# Three-Axis Ground Reaction Force Distribution during Straight Walking

**DOI:** 10.3390/s17102431

**Published:** 2017-10-24

**Authors:** Masataka Hori, Akihito Nakai, Isao Shimoyama

**Affiliations:** Department of Mechano-Informatics, Graduate School of Information Science and Technology, The University of Tokyo, 7-3-1 Hongo, Bunkyo-ku, Tokyo 113-8656, Japan; hori@leopard.t.u-tokyo.ac.jp (M.H.); nakai@leopard.t.u-tokyo.ac.jp (A.N.)

**Keywords:** ground reaction force (GRF), micro electro mechanical systems (MEMS), gait, walk, bipedal locomotion, 3-axis force sensor, shoe, force distribution

## Abstract

We measured the three-axis ground reaction force (GRF) distribution during straight walking. Small three-axis force sensors composed of rubber and sensor chips were fabricated and calibrated. After sensor calibration, 16 force sensors were attached to the left shoe. The three-axis force distribution during straight walking was measured, and the local features of the three-axis force under the sole of the shoe were analyzed. The heel area played a role in receiving the braking force, the base area of the fourth and fifth toes applied little vertical or shear force, the base area of the second and third toes generated a portion of the propulsive force and received a large vertical force, and the base area of the big toe helped move the body’s center of mass to the other foot. The results demonstrate that measuring the three-axis GRF distribution is useful for a detailed analysis of bipedal locomotion.

## 1. Introduction

Bipedal locomotion is one of the most remarkable features of humans. Many researchers have focused on the relationship between bipedal locomotion and the ground reaction force (GRF) [1,2,3,4]. Recently, force plates have enabled us to measure the three-axis resultant forces while walking on such a plate in limited laboratory settings. For walking action outdoors, only the normal force distribution can be measured by use of a piezoelectric film sensor inserted into the insole of a shoe [5,6,7,8,9]. However, researchers cannot obtain shear force distribution data from these methods, which is necessary for evaluating forward and lateral motion.

A large amount of the shear force distribution data with normal force distribution data obtained during walking is useful for analyzing bipedal locomotion in more detail. The force distribution data can be used to determine the locations at which large shear and vertical forces are applied during walking. This result will be helpful for optimizing the positions of stud pins in athletic shoes and for determining the best shoe cushion design. Thus, this knowledge is beneficial for shoe design, sports science, and medical treatment. Unfortunately, it is impossible to obtain the shear force distribution data from multiple steps using the current commercially available methods. Therefore, we fabricated a new force sensor and obtained the three-axis force distribution data. 

In this paper, we measured the three-axis GRF distribution during walking using a small three-axis force sensor attached to a shoe. The small three-axis force sensor was composed of rubber and a sensor chip fabricated using a micro-electro-mechanical system (MEMS). These force sensors were attached to the shoe after being fixed in a rubber frame without the use of a metal plate. Using this shoe–sensor combination, the three-axis GRF distribution was measured during straight walking on a treadmill. After measurement, the local features of the applied three-axis force under the shoe were analyzed.

## 2. Materials and Methods 

### 2.1. Three-Axis Force Sensor and Rubber Frame

**Figure 1:**
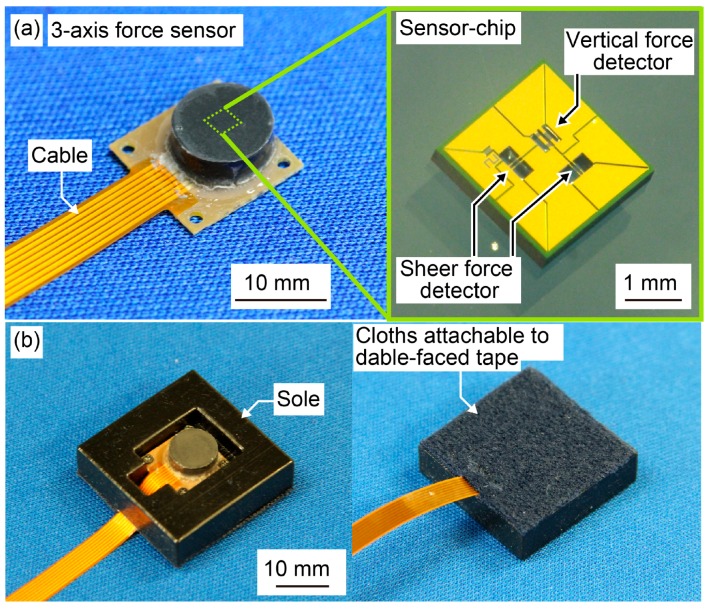
(**a**) Sensor chip and three-axis force sensor; (**b**) Top and bottom of the three-axis force sensor embedded in rubber material used as a sole.

The three-axis force sensor used for the measurements consisted of a sensor chip, a flexible cable, and thermoplastic rubber. The fabrication method, force detection principal, and a detailed feature of the sensor chip were described in [10]. Briefly, the dimensions of the sensor chip were 2 × 2 × 0.3 mm, and three pairs of Si-doped beams were fabricated on the sensor using a MEMS. These beams were used as the sensing elements for the applied three-axis force, and canceled out the effect of temperature change. This sensor chip was attached to the flexible cable substrate, and was electrically connected using conductive paste (XA-874, FUJIKURA KASEI Co., Ltd., Tokyo, Japan). After finishing the electrical connection, the sensor chip was covered with thermoplastic rubber (TSE3431-H(A) and TSE3431-H(E), Momentive Performance Materials Japan LLC, Tokyo, Japan). The covering rubber was 8 mm in diameter. The total dimensions of the three-axis force sensor were 11 × 11 × 3.5 mm, and the weight of the sensor was 0.6 g (Figure 1a). 

Before the force sensor was used for measurement, it was fixed in a rubber frame. The frame was made of rubber (TangoBlack, Stratasys Ltd., Rehovot, Israel) and was constructed using a 3D printer (EDEN260V, Stratasys Ltd., Rehovot, Israel). The Shore A hardness of the rubber material was 61, which is within the hardness range of the soles of commercially available shoes. The dimensions of the frame were 25 × 25 × 7 mm. To fix the three-axis force sensor, a 11.5 × 13 × 3.5 mm depression was made in the center of the sole, and a hole was created near the depression to allow the force sensor cable to pass through. The three-axis force sensor was fixed in the depression using clinchers. To attach the sensor to the shoe, a cloth was bonded to the backside of the frame using glue. The cloth was able to be detached via a hook-and-loop fastener (Figure 1b). The total weight of the three-axis force sensor and the frame was 15 g. 

**Figure 2:**
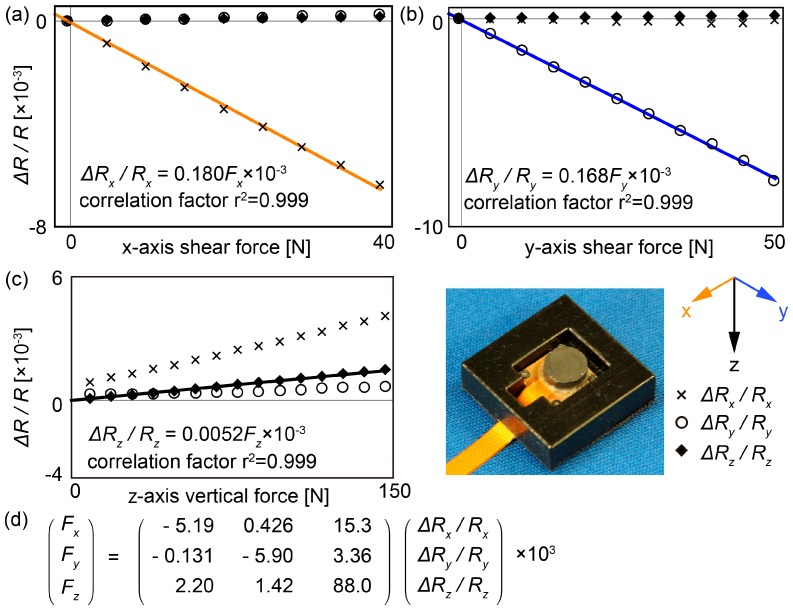
Outputs of the three-axis force sensor embedded in rubber material used as a sole. (**a**), (**b**) and (**c**) are the results when x-, y- and z-axis force were applied to the sensor, respectively. The markers in the graphs are plotted on the measured values. The solid lines in the graphs are fitting values obtained using the least-squares technique. (**d**) Equation of a force sensor to convert from resistivity changes to forces.

Vertical and shear forces were applied to the three-axis force sensor for calibration. A commercially available six-axis force sensor (SI-130-10, ATI Industrial Automation, Inc., Apex, NC, USA) was used as a reference device for the three-axis force sensor in the calibration experiment. The six-axis force sensor was fixed on the stage, and our three-axis force sensor that was fixed in the frame was attached to the top of the six-axis force sensor using a hook-and-loop fastener. An acrylic plate was fixed over the three-axis force sensor. In the calibration procedure, the stage was moved upward until our sensor touched the acrylic plate, and then vertical force was applied. Then, the stage was moved horizontally so that shear forces were applied to our sensor. The relationship between the applied force and the sensor outputs was determined from this calibration, and the result was used to measure the three-axis GRF distribution during walking (Figure 2). 

### 2.2. Measurement System

To measure the three-axis GRF distribution during walking, three-axis force sensors were combined with a shoe and circuits. 

**Figure 3:**
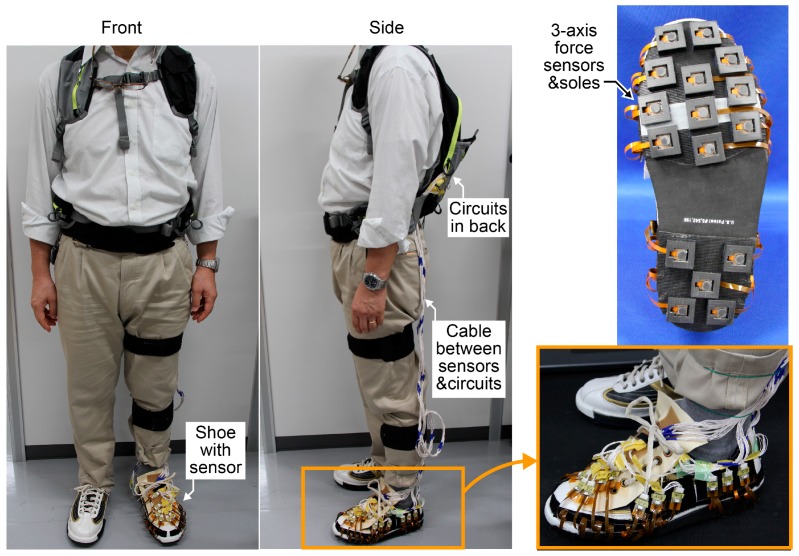
Shoe with three-axis force sensors and appearance of the person who wore the measurement system.

Commercially available shoes were selected for the measurement experiment (SST8, Dexter, MO, USA). The soles of these shoes could be attached and detached via the hook-and-loop fastener on the bottom surface. This feature was used to attach three-axis force sensors fixed in rubber frames to the left shoe. Rubber with the same thickness was attached to the sole of the right shoe. The shoes used were 26.0 cm in size. Eleven force sensors were used in the forefoot area, and five force sensors were used in the heel area. We set local coordinates on each sensor. When the subject stands with toes and heels together, the coordinate of the sensors in the heel area and the forefoot area rotates 10 degrees counterclockwise and 7 degrees clockwise with respect to the forward direction, respectively. The cables from the force sensors were fixed using a hook-and-loop fastener on the side surface of the shoes, and were connected with circuits through connectors and wires. The weight of the shoe with sixteen force sensors was 470 g. The circuits were carried on the subject’s shoulders with a backpack when the three-axis GRF distribution was measured (Figure 3).

To correct the force data during walking, a circuit was designed using an IC board (mbed NXP LPC1768, NXP Semiconductors N. V., Eindhoven, The Netherlands). On the circuit board, bridge and amplification circuits, low-pass filters, and A/D converters were mounted for the output channel of each three-axis force sensor. The voltage changes from the three-axis force sensors were measured using the bridge circuits and amplified 247 times, and 25 Hz were cut off by a low-pass filter. A Bessel filter was used as the low-pass filter, and its group delay was within 3% error from 0 to 20 Hz. After that, the signals were converted to digital data by 12-bit analog-to-digital (A/D) converters, gathered to the memory on the IC board, and sent to a PC using serial communication. The baud rate of the serial communication was 921.6 kbps, and the sampling frequency was 333 Hz. This circuit individually managed four force sensors, and we prepared four sets of circuits for measurement. Lithium batteries were used as the power supply. The dimensions and the weight of the four sets of circuits were 85 × 106 × 108 mm and 660 g, respectively. The total weight of the backpack with four sets of circuits, two batteries, and cables that was carried by the subject was 1100 g. 

We compared the sum of the sensor read and the body weight when the subject was conducting a static single-leg stand. The result of the former was 528 N, and the latter was 600 N. This indicates that the sensor read has around 10% error.

## 3. Experimental Results and Discussion

Using the proposed system, the three-axis GRF force distribution was measured when a subject walked on a treadmill. The subject was a healthy man whose height and weight were 168 cm and 61.2 kg, respectively. The walking velocity was a comfortable speed for the subject, and the analyzed data were averaged over the results from ten steps. The angle of the subject’s toe-off was 5 degrees counterclockwise. 

**Figure 4:**
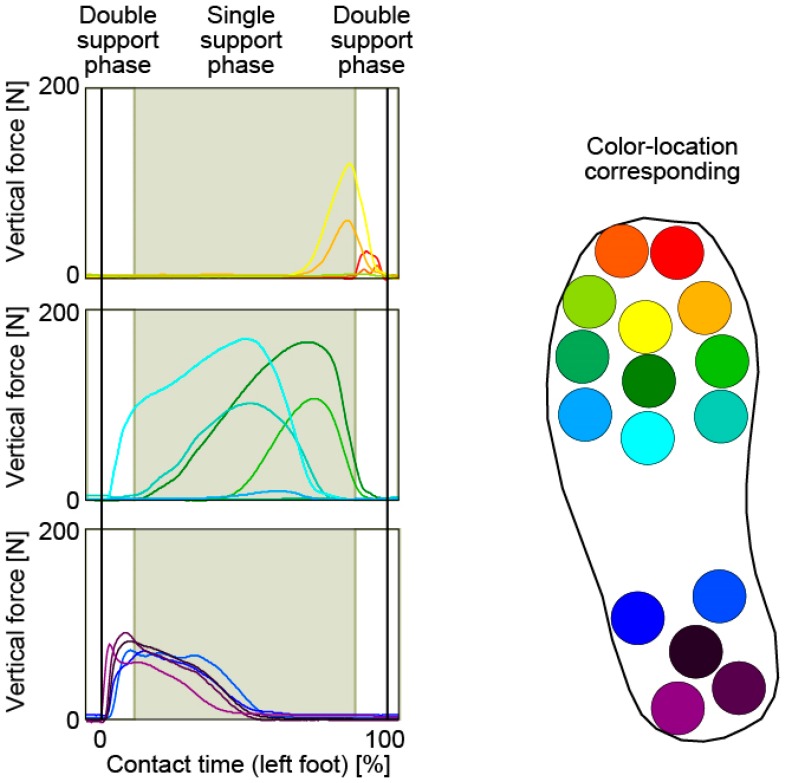
Change of vertical forces during the contact phase of the left foot.

Figure 4 shows the change in vertical forces during the foot contact phase. The durations of the single support phase and double support phase were calculated using the foot contact time and walking cycle. As shown in this figure, the contact area under the foot shifted from the heel to the toe. At the time of first contact between the left foot and ground, the entire heel area and the bottom of the forefoot area made contact nearly simultaneously. At the beginning of the single support phase, the base area of the toes contacted the ground gradually. Subsequently, the heel area lifted halfway off the ground through the single support phase. At the beginning of the right foot contact, the base area of all the toes were lifting off, and the toe area was in contact with the ground. 

**Figure 5:**
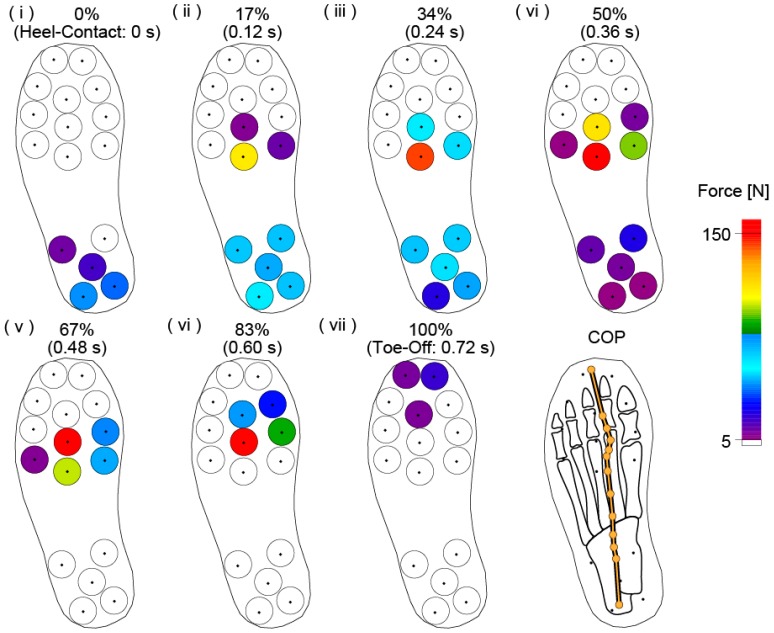
Result of the vertical force distribution measured by the proposed measurement system. COP: center of pressure.

Figure 5 shows the changes in the vertical force distribution over time during the foot contact phase and the center of pressure (COP) trajectory on a schematic of the relation between the location of the force sensors and the foot bone. The vertical forces are represented using a color scale: 0 N to 5 N is white, 5 N to 150 N gradually changes from purple to red, and over 150 N is red. The contact phase of the left foot was divided into seven parts, and the time of each data point is shown with the percentage of foot contact time. The time interval is 0.12 s, and the COP trajectory is plotted at 0.06 s intervals.

At the moment of left foot contact, the vertical force was distributed outside of the heel first and then received by the heel and forefoot (Figure 5i,ii). In this case, the largest vertical force was applied to the back area under the base of the third toe. After the beginning of foot contact, vertical forces at the forefoot were increasingly greater as the subject’s total mass moved forward (Figure 5iii,iv). Large vertical forces were applied to a small area under the third metatarsal. During the heel-off phase, vertical forces were primarily applied to the forefoot area of the first, second, and third toes (Figure 5v). During the toe-off phase, the area in which vertical forces were applied changed from the forefoot to the toe, and the forces in these areas became small (Figure 5vi,vii). This trend illustrates that the area under the third metatarsal is an important area during straight walking because of the magnitude of the vertical forces applied. Additionally, this area is likely at high risk of injury when this subject walks an excessively long distance because of the concentrated force. During this foot contact, the COP moved from outside of the heel to between the second and third toes. This trajectory is similar to the results of other research [11,12,13].

**Figure 6:**
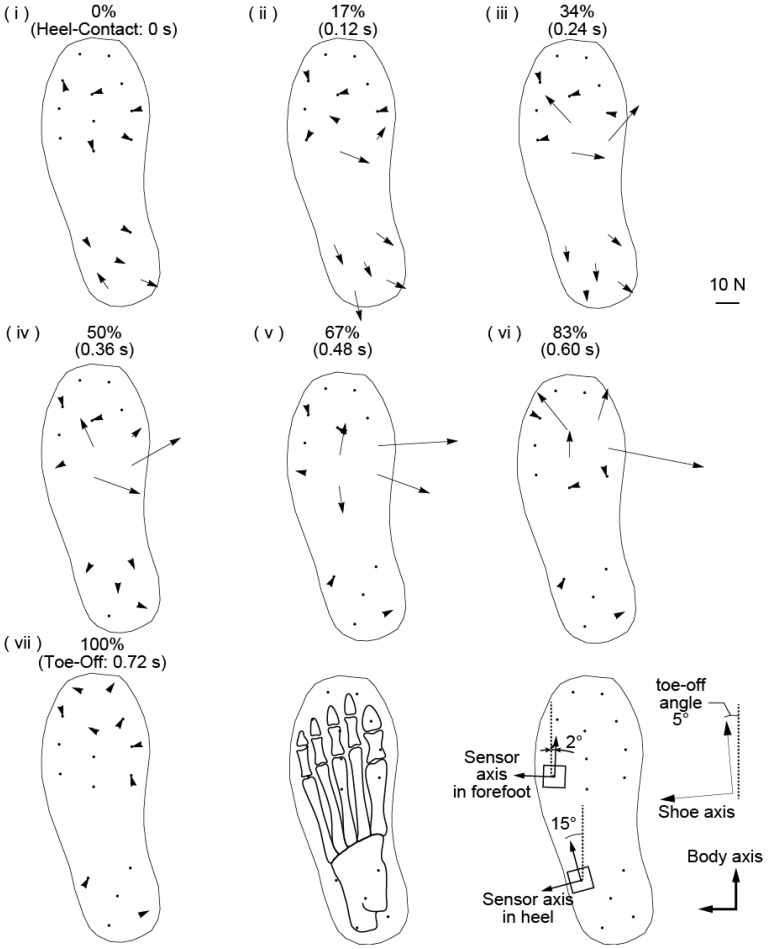
Result of the shear force distribution measured by the proposed measurement system.

Figure 6 shows the change in the shear force distribution over time during the foot contact phase and a schematic of the relation between the force sensor location and foot bone. The shear forces applied from the ground to the shoe are indicated by arrows from the center of each embedded force sensor. The strength of the shear force is represented by the length of the arrow, and the direction of the shear force is represented by the direction of the arrow. The time interval of the presented shear force data is 0.12 s. The contact phase of the left foot is divided into seven parts, and the time of each data point is shown as a percentage of the foot contact time. 

At the moment of left foot contact, a braking force was applied to the heel area and the center back area of the forefoot (Figure 6i,ii). A braking force of approximately 10 N was evenly applied to those areas. After the beginning of foot contact, the main area where the shear forces were applied moved from the heel to the forefoot. Although the shear forces applied to the heel area decreased in amplitude, the forces applied to the area under the base of the first, second, and third toes increased (Figure 6iii,iv). The shear forces in different areas had different features. The shear forces from the center back area of the first, second, and third toes were in the medial direction, indicating that this area plays a role in translating the center of mass from the left foot to the right foot. The shear force under the base of the third toe was in the anterior direction. Thus, the propulsive force is generated from this area. Additionally, these shear forces generated a torque in the counterclockwise direction along the vertical axis. During the heel-off phase, the area under the base of the big toe received a shear force toward the right foot (Figure 6v). The subject’s center of mass also moved toward the right foot due to this shear force and toward the center back area of the first, second, and third toes. During the toe-off phase, propulsive forces were applied under the first, second, and third toes (Figure 6vi,vii). Under the big toe, a propulsive force was applied in the medial direction. Conversely, the propulsive force under the third toe was applied in the direction of the long axis of the shoe. 

These results imply that the features of the shear forces during straight walking differ depending on the location of the sole for this subject. The heel area received braking forces early during straight walking, and the area under the first, second, and third toes generated propulsive forces at the end of walking. Although these features correspond well with previous knowledge of bipedal walking, three features from the forefoot area are observed halfway through straight walking [14]. The first feature was observed under the fourth and fifth toes. In this area, small shear forces were applied during straight walking, indicating that this area has a low degree of importance when the subject is walking straight. The second feature was observed under the base of the big toe. This area helped move the center of mass toward the other foot, and the generated shear force was directed to the right foot while the area was in contact with the ground. This result occurred because the momentum of the center of mass toward the left decreased and changed the momentum toward the right. This result indicates that the forefoot is separated into two areas: the area moving the center of mass forward, and the area moving it toward the other foot. The final feature was observed at the base of the third toe. Whereas the front area of the base of the toe generated a propulsive force, the back area of the base of the toe generated a braking force. This feature is assumed to be caused by foot motion in the shoe while walking. When a human is walking, the foot makes a gripping motion in the shoe. During this motion, the toes move as though they are scratching the ground, and the remaining area of the foot holds the ground. The joints between the metatarsal bones and phalanxes support this motion, and the area under these bones becomes the border where the direction of shear force changes. This observation is presumably why the directions of the shear forces are different between the front and back areas under the base of the third toe.

There are several advantages to our method. First, in our method, it is easy to increase the number of force sensors because of their small size. In walking research, several studies have focused on shoes with wearable force sensors [15,16,17,18]. In these studies, two-to-six three-axis force sensors were attached to the sole of the shoe, and the researchers attempted to measure the three-axis GRF distribution, even outside the laboratory. Although the results demonstrated the potential usefulness of these proposed measurement systems, the number of force sensors still requires improvement. When using only two-to-six force sensors, the distance between the sensors is rather large. Thus, a large space under the foot is covered by one force sensor, and the spatial resolution of the measured force data is low. This drawback could produce a deficient walking analysis of the three-axis force distribution. To increase the number of force sensors under a foot, where space is limited, the force sensors must be smaller. Our force sensors were fabricated using MEMS and miniaturized. Therefore, we can attach 16 force sensors to a shoe, which is more than twice the number of sensors used in previous studies. 

Second, in our method, even when many force sensors are attached to the shoe, the weight of the shoe is approximately maintained because only a small amount of metal material is used. In previous studies attempting to measure three-axis GRF distribution while walking, either the force sensors were made of metal or metal plates were used to fix the force sensors to the soles of commercially available shoes [15,16,17,18]. The use of metal could cause subjects discomfort, because such a hard and heavy material is not normally found in shoes. Additionally, if researchers want to attach many force sensors to a shoe, the weight of the shoe will become heavy and may affect the subject’s locomotion. To mitigate the drawbacks of using metal as much as possible, our fabricated force sensor was made primarily of rubber, which is in the hardness range of commercially available shoes. By using rubber, our force sensor is lighter than those used in previous studies. Thus, subjects who wear our shoes will experience under-foot sensations that are nearly the same as those before the force sensors were attached, and their locomotion will be largely unaffected.

According to its design, our method can measure a higher three-axis GRF distribution resolution, even outside the laboratory. Our shoes enable data to be collected even in locations where it is difficult to place force plates. The obtained three-axis distribution data cannot be collected using a pressure sheet. Compared to other proposed shoe measurement methods, the shape and weight of the shoe are further preserved, and our proposed method obtains the highest data resolution. 

An important observation from this experiment was that the individual features of the area under each subject’s sole can be discussed from the measured three-axis force distribution. From the resulting shear force distribution, there are several suggested roles for the forefoot area: a low contribution area, a propulsive force generating area, and a medial force generating area. Additionally, this result implies that the area under the third metatarsal is an area of concentrated applied vertical and shear force. These features observed during our walking experiment cannot be accepted as a common feature of human gait at this time. However, this measurement system can obtain a subject’s three-axis force features applied to definite sole areas, and the measurement of the three-axis force is meaningful for walking analysis. In the future, common three-axis force features during walking will be collected using more subjects. 

## 4. Conclusions

A three-axis GRF distribution during straight walking was measured using a shoe with three-axis force sensors. Sixteen small three-axis force sensors were fabricated with rubber and sensor chips. The weight of the three-axis force sensor with a rubber frame was 15 g. The force sensors were attached to the removable sole of the left shoe using a hook-and-loop fastener. The data from these 16 force sensors were collected at a sampling frequency of 333 Hz. Our shoe measurement system was used to measure the three-axis GRF distribution during straight walking on a treadmill. From the experiment, extensive three-axis-GRF-distribution data were obtained for the first time. The local roles under the foot were revealed by analyzing the distribution data: the heel received a braking force during the foot contact phase, the base of the fourth and fifth toes applied a small three-axis force during straight walking, the base of the second and third toes applied a strong vertical force and propulsive force from the mid-standing phase to the take-off phase, and the base of the big toe applied a medial force from the mid-standing phase to the take-off phase. These results indicate that the applied three-axis GRF force differed at different locations under the foot. Therefore, measuring and analyzing the three-axis GRF distribution during walking has enormous significance, and may yield new insights for walking researchers. In the future, we believe that this measurement system and the results of this study will be crucial for further detailed analyses of bipedal locomotion.

## References

[1] Elftman H. (1934). A Cinematic Study of the Distribution of Pressure in the Human Foot. Anat. Rec..

[2] Elftman H. (1938). The Measurement of the External Force in Walking. Science.

[3] Rajala S., Lekkala J. (2014). Plantar shear stress measurements—A review. Clin. Biomech..

[4] Jung C.K., Park S. (2014). Compliant Bipedal Model with the Center of Pressure Excursion Associated with Oscillatory Behavior of the Center of Mass Reproduces the Human Gait Dynamics. J. Biomech..

[5] Stolwijk N.M., Duysens J., Louwerens J.W.K., Keijsers N.L.W. (2010). Plantar Pressure Changes after Long-Distance Walking. Med. Sci. Sports Exerc..

[6] Cong Y., Lee W., Zhang M. (2011). Regional Plantar Foot Pressure Distributions on High-Heeled Shoes-Shank Curve Effects. Acta Mech. Sin..

[7] Decker L., Houser J.J., Noble J.M., Karst G.M., Stergiou N. (2009). The effects of shoe traction and obstacle height on lower extremity coordination dynamics during walking. Appl. Ergon..

[8] Lieberman D.E., Venkadesan M., Werbel W.A., Daoud A.I., D’Andrea S., Davis I.S., Mang’Eni R.O., Pitsiladis Y. (2010). Foot Strike patterns and Collision Forces in Habitually Barefoot versus Shod Runners. Nature.

[9] Dxon S.J., Collop A.C., Batt M.E. (2000). Surface Effects on Ground Reaction Forces and Lower Extremity Kinematics in Running. Med. Sci. Sports Exerc..

[10] Takahashi H., Nakai A., Thanh-Vinh N., Matsumoto K., Shimoyama I. (2013). A Triaxial Tactile Sensor without Crosstalk Using Pairs of Piezoresistive Beams with Sidewall Doping. Sens. Actuators A: Phys..

[11] Orlin M.N., McPoil T.G. (2000). Plantar Pressure Assessment. Phys. Ther..

[12] Hatala K.G., Dingwall H.L., Wunderlich R.E., Richmond B.G. (2013). The relationship between plantar pressure and footprint shape. J. Hum. Evolut..

[13] Bruening D.A., Cooney K.M., Buczek F.L., Richards J.G. (2010). Measured and estimated ground reaction forces for multi-segment foot models. J. Biomech..

[14] Rueterbories J., Spaich E.G., Larsen B., Andersen O.K. (2010). Methods for gait event detection and analysis in ambulatory systems. Med. Eng. Phys..

[15] Veltink P.H., Liedtke C., Droog E., van der Kooij H. (2005). Ambulatory Measurement of Ground Reaction Forces. IEEE Trans. Neural Syst. Rehabil. Eng..

[16] Liedtke C., Fokkenrood S.A.W., Menger J.T., van der Kooij H., Veltink P.H. (2007). Evaluation of Instrumented Shoes for Ambulatory Assessment of Ground Reaction Forces. Gait Posture.

[17] Liu T., Inoue Y., Shibata K. (2010). A Wearable Ground Reaction Force Sensor System and Its Application to the Measurement of Extrinsic Gait Variability. Sensors.

[18] Liu T., Inoue Y., Shibata K. (2010). A Wearable Force Plate System for the Continuous Measurement of Triaxial Ground Reaction Force in Biomechanical Applications. Meas. Sci. Technol..

